# Exploring the Perceptions of and Experiences with Traditional Foods among First Nations Female Youth: A Participatory Photovoice Study

**DOI:** 10.3390/ijerph17072214

**Published:** 2020-03-26

**Authors:** Rebecca Hanemaayer, Kimberley Anderson, Jess Haines, Kitty RLynn Lickers, Adrianne Lickers Xavier, Kelly Gordon, Hannah Tait Neufeld

**Affiliations:** 1 Department of Family Relations and Applied Nutrition, University of Guelph, Guelph, ON N1G 2W1, Canada; rhanemaa@uoguelph.ca (R.H.); kimberle@uoguelph.ca (K.A.); jhaines@uoguelph.ca (J.H.); 2 Six Nations Health Services, Ohsweken, ON N0A 1M0, Canada; klynnlickers@sixnations.ca (K.R.L.); kgordon@sixnations.ca (K.G.); 3 Department of Indigenous Studies, McMaster University, Hamilton, ON L8S 4L8, Canada; Xavier@mcmaster.ca; 4 School of Public Health and Health Systems, the University of Waterloo, Waterloo, ON N2L 3G1, Canada

**Keywords:** traditional foods, Indigenous foods, traditional food systems, Indigenous food sovereignty, traditional knowledge, Indigenous youth, southern Ontario

## Abstract

Traditional foods contribute to the health and well-being of Indigenous Peoples. Many Indigenous Peoples within Canada have expressed a desire to consume more traditional foods; however, there are a number of barriers to doing so. Southern and urban communities face unique challenges associated with traditional food consumption. To address these concerns and build on community interests in a Haudenosaunee community in Southern Ontario, a participatory research project was initiated. This community-based study utilized Photovoice methodology to explore the perceptions of and experiences with traditional foods among local youth. Participants ranging in age from 15–22 (*n* = 5) took photos of their local food environments, including locations where foods were acquired, consumed, prepared, or shared during two seasons of the year. Semi-structured interviews were conducted to collect participants’ stories behind 8–10 self-selected images. A thematic analysis was subsequently utilized to identify patterns and themes illustrated by the photos and interview content. The youth conveyed contextual understandings of traditional foods and a preference for these items, despite their limited consumption, preparation or harvesting of these foods. The youth also identified the important influence of families and communities on their individual perceptions and experiences with traditional foods. Recommendations to reduce barriers to traditional food choices among youth are made.

## 1. Introduction

Land and food are integral to the health and well-being of Indigenous Peoples and are important proponents of physical, cultural, nutritional, and spiritual well-being [1,2,3,4,5]. The word ‘traditional’, used throughout this article, varies in meaning to different people. To some, this term depicts the circumstances that existed in a pre-settler society uninterrupted by colonization, whereas others use the word ‘traditional’ to describe Indigenous Peoples’ cultures in the present context. Most commonly, traditional foods are defined as those that are available to a particular Indigenous culture from the local natural environment, including both plant and animal species [6,7,8]. Traditional Indigenous foods are embedded within broader, multifaceted traditional food systems, which encompass “the sociocultural meanings, acquisition, processing techniques, use, composition, and nutritional consequences for the Indigenous Peoples using these foods” [3] (p. 2). As such, traditional foods and food systems can vary greatly, based on factors such as geography, seasonality, and cultural group [6,8]. Though Indigenous groups continue to identify the value of traditional foods in contributing to holistic well-being, traditional food intake among Indigenous Peoples in Canada has gradually declined since their first contact with European settlers [4,8].

The vast majority of existing Canadian studies that pertain to traditional foods have sought to quantitatively investigate traditional food consumption behaviors among Indigenous Peoples. Results conveyed generally indicate that while many Indigenous adults report consuming traditional foods, most do not habitually engage in traditional food practices [9,10]. These studies have primarily taken place in northern and remote Indigenous communities; however, a recent national study on the diets and health of First Nations adults found that traditional food consumption was highest in more northern and western ecozones [9]. In contrast, the lowest consumption of traditional foods was found among those living in Prairie ecozones, Mixedwood Plains, and Atlantic Maritimes. The Mixedwood Plains ecozone covers the most southern region of Canada. Similarly, another national study also found the habitual consumption of traditional foods to be lowest in southern and urban First Nations communities compared to those that are more rural and remote [10]. From 2008-2010, only 29.5% of First Nation adults living in these southern regions regularly consumed wild meats. Even fewer (18.1%) regularly consumed plants harvested from the local environment [10].

Though there appears to be a limited consumption of traditional foods among Indigenous Peoples living in Canada, these trends are not related to a lack of desire to consume these foods. In fact, 77% of First Nation adults living across Canada would like to consume traditional foods more often [9]. Despite this desire to consume more traditional foods, however, there are a number of barriers to doing so. The gradual decline in traditional food consumption among Indigenous Peoples within Canada is also a global phenomenon known as the nutrition transition [4,7]. Around the world, local traditional food systems and practices have been threatened and eroded by structural impacts, including processes of colonization. Indigenous Peoples have been forced to rely more and more on dominant industrialized food systems [4].

A limited number of studies have explored the complex determinants of traditional food consumption. As traditional food systems are land-based, access to land and water has been identified as a significant barrier to acquiring or accessing traditional foods [3,4,7,8,11]. Notably, processes of environmental dispossession of Indigenous Peoples from their traditional territories have negatively impacted the accessibility, availability, and use of traditional food sources [3,7,12]. In addition, those living in urban areas tend to have reduced access to land and water where traditional foods naturally exist [2,7,11]. Environmental and climate changes are widely cited factors that have reduced the ability of Indigenous Peoples to acquire and rely on traditional food sources [4,7,9]. Many are also concerned about environmental contaminants reducing the safety of traditional food sources [7,8,9,13]. The cost of the equipment needed to acquire traditional foods and the time required to do so have also been identified as economic barriers [4,7,8,9,11]. Colonial policies and practices have also reduced opportunities for knowledge transmission, contributing to a loss of traditional knowledge related to the utilization of traditional foods over time [3,4,7,14]. Political factors or determinants underlie many of the barriers described in the literature. Government legislation have historically limited, for example, where Indigenous Peoples are allowed to hunt, fish, and gather traditional foods [4,7,8,9,11]. Most notably, the colonial government’s implementation of the residential school system has had a detrimental impact on traditional food systems and cultural practices [7,14].

At the individual level, food preferences influence the choice to consume traditional foods [3,4]. The influence of family, specifically the availability of traditional foods in the home environment, may impact the preference of traditional foods for children and youth [4,11,13]. Access to traditional foods is also inextricably tied to social environments, as traditional foods are often shared through personal connections. However, those living in urban areas often have reduced opportunities for sharing, as a result of limited connection to family and community members [2,3,7,11].

There are a number of significant limitations to the existing literature and research that has taken place thus far on traditional food systems and practices in Canada. Despite findings that Indigenous Peoples living in southern and urban regions are among the lowest habitual consumers of traditional foods, most existing literature exploring traditional food practices has focused on the context of northern and remote Indigenous communities [15,16,17,18]. It is also clear that there are many barriers to traditional food consumption, with considerable and unique challenges experienced among those living in southern and urban areas [2,7]. With increasing migration to urban centres and more than half of the Indigenous population living off-reserve, it is important to consider barriers to traditional food consumption in these contexts [2,19]. In addition, the existing body of evidence on traditional food systems has primarily focused on Indigenous adults. Youth and children make up a significant part of the Indigenous population, with over half of Indigenous Peoples within Canada being under the age of 25 [19]. Most studies investigating dietary patterns among Indigenous Peoples within Canada have found that traditional food consumption increases with age [9,14,15,20]. The participation of youth is therefore necessary to revitalize and sustain traditional food systems and practices for generations to come [2,21]. Finally, most research investigating traditional food patterns has been quantitative, investigating the amount of traditional foods consumed, rather than exploring the determinants of traditional food consumption. Research investigating the preferences and attitudes toward traditional foods is also severely lacking [21].

The present study was developed to address these significant gaps in the literature, and build on community interests, with the aim of exploring the perceptions of and experiences with traditional foods among youth living in a Haudenosaunee community in southern Ontario. The objectives of this study were to: (a) explore how First Nations youth understand, define, and value traditional foods, (b) explore the traditional food experiences of First Nations youth, including experiences in growing, acquiring, preparing, consuming, and learning about traditional foods, (c) investigate the determinants of everyday and traditional food choices among First Nations youth, including perceived facilitators and barriers to the availability, access, and use of traditional foods and traditional knowledge, and (d) investigate perceived opportunities that would improve the availability, access, and use of traditional foods and traditional knowledge individually and in the community. This paper presents the findings from this study pertaining to participants’ thoughts, beliefs, and experiences with traditional foods.

## 2. Materials and Methods

The study was funded through the Canadian Institutes for Health Research (CIHR) and is embedded within a larger research project, investigating sources of traditional foods and knowledge in urban and reserve communities in southwestern Ontario. This portion of the research took place in a Haudenosaunee community in southern Ontario, and resulted from a research partnership with the local health centre. In recent years there had been a growing interest among community members in reconnecting to traditional Haudenosaunee culture and foodways (Haudenosaunee refers to “The People of the Longhouse”, comprised of Mohawk, Cayuga, Oneida, Seneca and Onondaga Nations, and Tuscarora Nations) [5]. This growing interest in traditional food systems has been supported and enhanced by the recent introduction of several community food initiatives, aimed at increasing local access to fresh foods and traditional foods. The study was initiated by community members and took a participatory approach that involved those engaged in the work as partners and decision-makers in each stage of the research process.

Community-based participatory research (CBPR) formally aims to minimize researcher-participant power dynamics, build community capacity, foster trusting relationships, and develop a community sense of ownership of the research project, in order to work toward social justice and change [22,23]. The overall idea and vision for the present study arose from conversations with health services staff, who continued to be champions throughout the ongoing development of this research project. A research assistant was hired locally, who supported the implementation of this study in a number of important ways, such as engaging other community members through the organization of a Community Advisory Group who met four times per year to make decisions regarding how the research was implemented and results disseminated.

Photovoice methodology was utilized as part of the study. Photovoice is a flexible arts-based methodology where participants take photographs to capture their views and experiences related to research questions and participate in interviews or sharing circles to share the stories behind their photos [24]. Consistent with the principles of CBPR, Photovoice projects help minimize researcher-participant power dynamics, by providing participants with the autonomy to complete photo assignments on their own time and choose the subject and meaning of the photographs taken [23,24]. Photovoice provides a unique pathway for exploring topics and issues of interest to community members [22,24]. Photovoice has been identified as a culturally appropriate, methodologically rigorous, and ethically sound methodology to use in research with Indigenous Peoples [22,24]. Photovoice has also been identified as a particularly useful methodology for involving youth in research [25].

Individual semi-structure interviews were used to collect rich and detailed information from participants. A semi-structured interview guide was developed in collaboration with the research team, and the Community Advisory Group. The guide included open-ended questions regarding participants’ personal and family food choices, as well as their perceptions and experiences with traditional foods. A series of questions were also included to collect the stories behind each of the participant photos. The interview guide was piloted and shared with the Community Advisory Group, prior to beginning participant interviews.

Ethical approval was received from the community Research Ethics Board, as well as the University of Guelph’s Research Ethics Board (REB #: 17-03-034), before advertising for participants. This study was promoted at community events and programs, via social media, posters, and word-of-mouth. Five young people living in the community were recruited and participated in this study from February to July 2019, with three participants taking photos during the winter months and two over the summer. A brief description of these participants is provided in Table 1. Pseudonyms were chosen at random by the researcher. Of note, all participants self-identified as female and were between the ages of 15–22 at the time of their participation.

Each participant met with the first author and community research assistant at a time and location of their choosing, to provide an overview of the research project, obtain informed consent, and complete photo release forms. At this time, youth were also provided with training on how to use the digital cameras provided. Participants were instructed to take as many photos as they would like of their local food environments, including anywhere foods were consumed, acquired, grown, prepared, or shared. In order to protect privacy, youth were asked not to capture any human subjects in their photos. Participants chose 8–10 of their photos to discuss in their individual interviews. The interviews were audio-recorded with permission, using an audio-recorder and iPod voice recorder. The interviews lasted between 64 to 79 min. After their interviews, participants were each given a $50 Visa gift card for their participation and asked to indicate their favourite photo, so that we could provide them with a framed copy.

Following the initial data collection, the first author transcribed the interviews verbatim. Participant names and other potential identifying information, such as family names and locations, were removed from interview transcripts with feedback from the participants. A thematic analysis was chosen to analyze the content of the interview transcripts and photos [26]. The qualitative research analysis software, NVivo, was used to manage and analyze the data collected, including photos and interview transcripts. Braun and Clarke’s [26] six-stage process for thematic analysis was used to guide the process of analyzing data. Following this process and using the NVivo software, interview transcripts were coded and later organized into themes that related to the research objectives. The photos collected were also analyzed for themes. Photos were organized and saved with their corresponding interview transcripts, so that they could be analyzed at the same time. Photos were organized into general categories to identify types and sources of food captured, how and with whom food was consumed, and places of food consumption. After assigning these codes to each photo, it was possible to identify overarching and recurring themes related to the content of the photos.

## 3. Results

The results presented in this paper pertain to a portion of the overall study results. This paper focuses on describing findings regarding participants’ thoughts, beliefs, and experiences with traditional foods. Interviews with participants included specific questions regarding their personal and family food choices, their perceptions of traditional foods, and their experiences of traditional foods. The primary focus of the interviews was on collecting the stories behind participants’ photographs of their food environments. Some, but not all, participants continued to discuss traditional foods in their photo discussions. Four emergent themes relating to traditional foods were identified. These main findings were that traditional food understandings are contextual, youth generally had limited knowledge and experience with traditional foods despite a preference for these foods, and that families and the community impacted their perceptions and experiences of traditional foods. These themes are discussed in detail in this section, with the use of direct quotations and participant photos to illustrate these findings where possible.

### 3.1. Traditional Food Understandings are Contextual

At the start of their interviews, participants were asked to identify what foods they thought of as “traditional.” It became evident that participants had diverse and conflicting understandings of what the term ‘traditional foods’ encompassed. Some foods were collectively identified by all or most participants as traditional foods, while other foods were mentioned by select participants only. For example, all participants considered corn soup and deer meat as traditional foods. Four participants included strawberries or strawberry juice and ‘mush.’ (Wild game and harvested plants such as corn and strawberries have been recognized by local health authorities as traditional foods to the community, as they are known to have existed within Haudenosaunee Territories prior to first contact [5]; mush is a traditional Haudenosaunee dish prepared by boiling ground white corn [27]). Less commonly identified traditional foods included scones and other baked goods. Three participants identified, for example, Indian cookies, Indian donuts, and scones as traditional foods. (These types of baked goods described by participants are not generally classified as traditional foods, due to their use of ingredients commonly referred to as the “five white gifts” (flour, salt, sugar, milk, and lard), which were initially introduced to Indigenous Peoples in the form of government-issued rations [5]). Finally, cornbread was identified by two participants.

**Figure 1:**
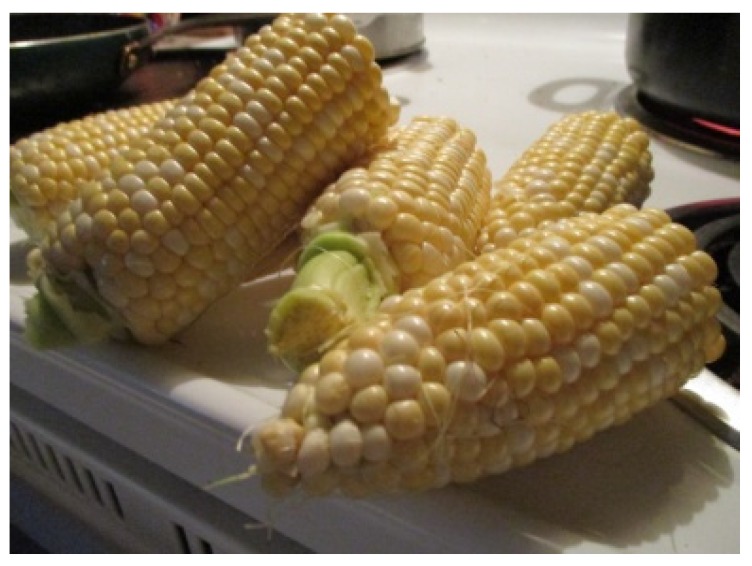
Margaret, 22.

A traditional food classification that held some contention among participants was scone (a prepared bread). While two participants did not mention scone at all, Grace identified how she would not consider the bread to be traditional. She stated, “I wanna say scone, but scones aren’t traditional.” There were several other examples from the interviews that highlighted how participants conveyed their unique understandings of traditional foods. For instance, during Georgia’s interview, her mother was present for the introductory questions. Georgia disagreed with her mother’s suggested classification of wild turkey as a traditional food. Grace provided another example and indicated her preference for corn soup made with white corn, which she identified as a traditional food, compared to yellow corn soup. On the other hand, it was clear that Margaret thought of yellow corn as traditional when she discussed the photo depicted in Figure 1. She explained, “[this photo shows] we still kind of eat traditionally.”

Participants also described how the ingredients and preparation techniques used in procuring specific dishes influenced their nutritional quality and whether they classified them as traditional. For example, Margaret stated: “strawberry juice… I mean I guess it’s traditional, but not like the way they make it now.” She went on to discuss in more detail that the methods of traditional food preparation have changed over time. She explained, “I like ‘em [traditional foods]. I just don’t think that they’re really… like genuine anymore… like people do stuff to it and things like that and that’s- it’s fine ‘cause we all have to adapt.” Two participants discussed how preparation methods influenced the healthfulness of traditional dishes. For example, Melody stated, “’cause they [traditional foods] are really good for you… but it kind of depends on how you cook it as well.” While Melody regarded many traditional foods as being nutritious, Georgia considered most traditional dishes to be unhealthy. She explained:

“I think they’re too fat [traditional foods]… well most of them… ‘cause the corn soup has like a lot of salt in it right? I don’t know it’s just salty to me. Indian donuts, it has like a lot of grease. Oh ham scone, that’s greasy.”

### 3.2. Preference Despite Limited Knowledge and Consumption

Another theme identified in the study results was that participants had limited knowledge and consumption of traditional foods, despite a preference for these items. After defining what traditional foods meant to them, participants were asked to share their personal thoughts and experiences with traditional foods. Interestingly, though all women talked about their enjoyment of and the taste of traditional foods, there were very few pictures of these foods. When asked about their experiences with traditional foods, it became apparent that the majority of participants’ experiences were related to the consumption of traditional foods, with fewer having experiences growing, gathering, or preparing them.

#### 3.2.1. Preference for Traditional Foods

During their interviews, participants collectively expressed preferences for the taste of what they considered to be traditional foods. The first author asked youth questions about their thoughts on traditional foods early on in their interviews, before moving on to discuss each of their photos. Two youth spoke about traditional foods before they were asked about them. One young person, Margaret, highlighted traditional dishes as being among her favourite foods. She said, “fajitas I think is my favourite… and then I don’t know. I like corn soup… If I could I would eat like fajitas or corn soup or mush like every day.” The other participant, Melody, spoke with pride of preparing a traditional dish in her foods class at school:

“I love that [foods] class… and then like Iron Chef competition which my group ended up winning… actually my group was the Native group. There was four of us… so we made umm lemon and garlic marinated venison with potato slices and orange and kale salad. It was really good.”

All participants demonstrated an enjoyment of the taste of traditional foods in their discussions. Maria shared, “love ‘em [traditional foods]. You can’t go wrong with them… it’s just- it’s good”. Similarly, Melody told us, “but yeah I really like them [traditional foods] like all of it tastes good to me”. Georgia described how she enjoyed traditional foods on certain occasions: “[Traditional food] tastes good… when I’m in the mood”. Finally, Grace said this: “Oh man they’re so good [wild meat pepperettes]”.

#### 3.2.2. Limited Traditional Food Knowledge

**Figure 2:**
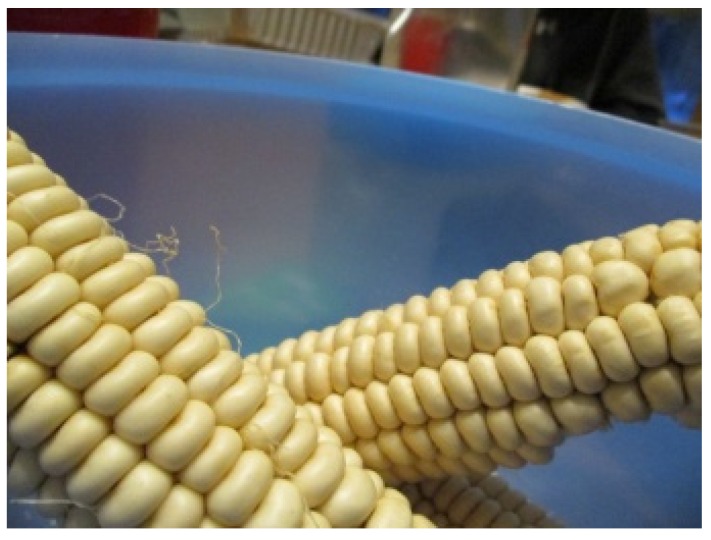
Grace, 15.

In their discussions of their traditional food experiences, it became apparent that participants had limited experiences in growing, acquiring, and preparing traditional foods. In particular, there were no participants who talked about personal experiences growing or harvesting traditional foods. Maria explained her lack of personal experience growing traditional foods when she said, “we don’t really do gardening. I guess the only one that really gardens in my family on my mom’s side is my uncle.” While four of five women did describe experiences in gathering traditional foods, these experiences appeared to be on limited occasions, rather than a regular occurrence. For example, Maria shared: “I went strawberry picking once.” Another participant, Grace, illustrated that she had limited knowledge of where traditional foods come from, when describing her photo of white corn (Figure 2). She said: “I don’t know where people get white corn from… it’s just kind of there.”

While most participants did report some experience preparing traditional foods, similar to their growing and gathering experiences, these were on limited occasions. Their experiences were generally viewed as enjoyable. As described earlier, Melody shared with pride how she prepared a traditional dish for a competition in her high school foods class. Another participant, Grace, explained how she had enjoyed observing traditional food preparation: “but like seeing it [traditional foods] get made and like eating it is kind of cool.” Grace went on to describe how she had made Indian cookies on her own: “I don’t always make them but like I tried to make them once and it went pretty alright.” Maria also shared her experiences preparing traditional foods: “I’ve helped cook ‘em… especially at my grandma’s… I’d help her make scones or just bread or the Indian cookies or Indian donuts too.”

These few experiences preparing traditional foods demonstrate how, though participants enjoy the taste and have a preference for traditional foods, they may have limited knowledge and skills related to how to prepare them. Margaret directly described how her lack of experience preparing traditional foods limited her consumption of dishes that she enjoyed by stating: “I really like corn soup but I don’t get to eat it a lot because I don’t know how to make it… It should be fairly easy because it’s soup like how hard can it be… I’ve just never tried to do it.” She went on to describe how her limited food skills were a barrier to consuming more traditional foods: “I just don’t like know how to make things right… I would [like to eat more traditionally]… I think we’re too absorbed in Westernized culture… colonized.”

#### 3.2.3. Limited Habitual Consumption

Though participants reported that most of their traditional food experiences involved consuming them, it also became apparent that they did not regularly consume traditional foods. One piece of evidence to support this was the lack of photos taken of traditional foods. While each participant shared 8-10 photos of food in their everyday lives, only three photos, taken by two participants, were identified by participants as traditional foods. Furthermore, the times participants did report consuming traditional foods were primarily at special occasions, rather than habitually or on a weekly or daily basis. While one participant, Maria, did speak about consuming traditional dishes regularly within a typical week, the remaining four female youth did not share this practice.

#### 3.2.4. Interest in Learning

While participants had limited experiences that involved growing, acquiring, or preparing traditional foods, three participants indicated a desire to learn more. As one example, Grace stated: “I haven’t seen them make it [cornbread]… I wanna see how it’s made.” Melody passionately described how she believes there should be more opportunities to learn about and celebrate traditional foods in the community:

“There’s not enough recognition for it… [traditional foods] should be celebrated more… I’d say like more introduced I guess like to people… it’s like ‘oh yeah those are just are traditional foods’ not ‘check out this, we have so many stories about it.’”

While several women described wanting to learn more, barriers to learning and preparing traditional foods were also brought up. Melody explained during her interview how there were limited opportunities for people to learn about traditional foods: “unless you really ask someone- like you were super like, ‘I really want to learn this’ - you would have to go find someone [to learn about traditional foods].”Another participant, Margaret, who expressed a desire to consume more traditional foods shared, “I just don’t have the time right now [to learn].

### 3.3. Importance in the Community

**Figure 3:**
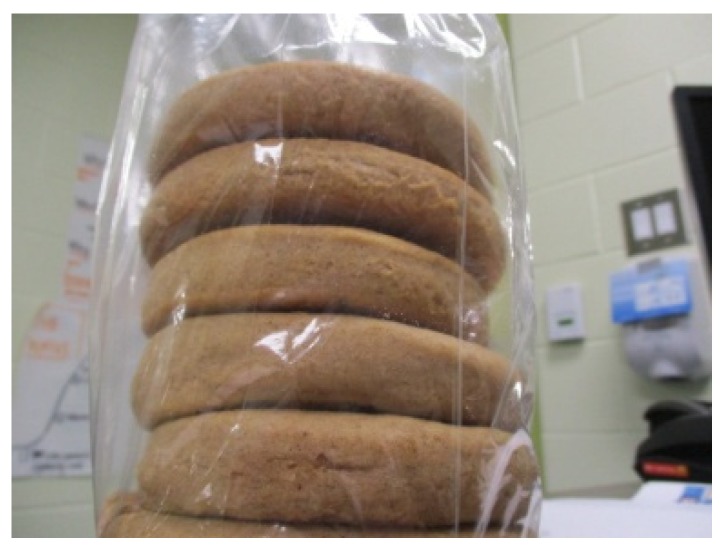
Grace, 15.

Another major theme that arose during participant interviews was the importance of traditional foods in the wider community. Female youth described how traditional foods are well known among community members. Grace described a collective understanding and appreciation of traditional foods among those living on the reserve, when she spoke about her photo of Indian cookies (Figure 3): “they’re my favourite cookie that’s like tied to uh the Rez… because like everybody knows what Indian cookies are.”

**Figure 4:**
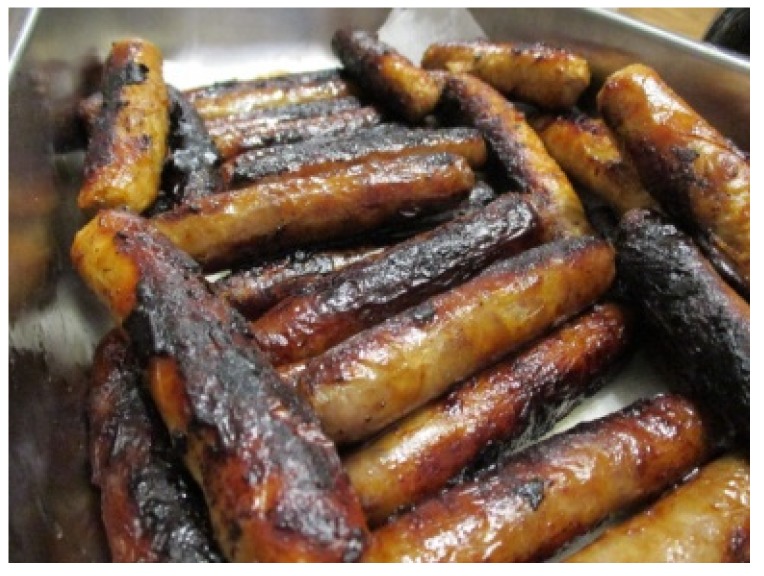
Grace, 15.

When describing her photo of white corn (Figure 2), she also described how dishes are considered traditional, not just in her family, but in the context of the broader community as well. She said, “I like corn soup. Corn soup’s traditional. Corn soup everybody eats.” In another photo she took at an extended family celebration (Figure 4), Grace explained the photo was meant to convey the importance of wild meat for the community. She said, “meat is a big thing around here like game and stuff.”

#### 3.3.1. Community Events

While most participants reported that they did not regularly consume traditional foods, all described community events and gatherings as a time that they were able to eat these foods. Several participants brought up the celebration of a new year in their community. Georgia, for example, explained how she participated in this annually and described this celebration: “It’s just people making cookies and giving them out cause it’s a new year… you say ‘happy new year’… and then they give you a treat and then you go on to the next house. It’s like Halloween.” Grace also referred to this celebration when she talked about her photo of Indian cookies (Figure 4). She described her personal experience with the time of celebration when she recalled her, “grandma making me stay up till like 3 o’clock in the morning making cookies and donuts for people to come around and get them.”

Participants described other community events as times when they were able to experience and enjoy traditional foods. Maria stated how: “any kind of event too like they can have it [traditional foods].” Georgia described other events where traditional foods would be served: “people have ‘em for birthday parties or… Pow Wows… longhouse.” Margaret talked about community events where traditional foods were available:

“Just like when people are selling it at like vendors and stuff like at Pow Wow… or like for fundraisers or whatever… I don’t know like at events or whatever ‘cause like people don’t sell them at like food booths or anything that I’ve noticed anyway”.

For several women, community events were, in fact, the main place they consumed traditional foods. Melody stated: “longhouse is the only time I really eat deer meat.” Similarly, Margaret shared that the main times she got to eat traditional foods were at special events: “traditional foods just kind of remind me of funerals cause that’s like where you have it the most… that’s where I have it the most… or longhouse.”

#### 3.3.2. Traditional Elementary Schools

Traditional schools in the community (privately run traditional schools in the community offer traditional language immersion programs and unique curriculums that aim to preserve local traditions and cultures, while preparing students to pursue further education or opportunities in the workforce [28]) were another place that two women reported learning about and experiencing traditional foods. Grace referred to her elementary school education as a formative influence on her current knowledge of traditional foods: “kind of like elementary school because they try to get that into your brains because when you go out to high school like you don’t learn that kind of thing.”Another participant, Melody, described how attending a traditional school was a significant source of traditional food knowledge for her and how, outside of school, there were limited opportunities to learn about traditional foods:

“You wouldn’t know about it [if you didn’t attend a traditional school]… you would hear stories about it [traditional foods] if you’re really traditional but like other than that… like nobody knows how much we’ve contributed to the food menu like it’s crazy”.

Grace explained what she learned about traditional foods as an elementary school student when she said: “so we would’ve like talked about the certain food and like we would’ve went into if it was used in a ceremony… or we would’ve talked about when it’s like supposed to be eaten.”As Melody described her traditional food experiences, she noted that her experience gathering and preparing traditional foods had mostly been through school activities:

“I went to like a really traditional school so we would always make mush like corn soup, so I immediately think of those things when I think of traditional foods. Once again another field trip we went corn picking at our school”.

### 3.4. Family Influence

Another theme that arose from participant discussions was the influence of family on their traditional food experiences, consumption, and knowledge. Participants identified their immediate family as influencing the amount of traditional foods they habitually consumed. Four of the five women reported that they did not regularly have traditional dishes served at home. However, one participant, Maria, identified her immediate family to be her main source of traditional foods, when she stated: “basically my family [is the source of traditional food]—‘cause just come home and all of a sudden there’s corn soup ready and I’m just like ‘okay that’s what we’re having’.” Grace, on the other hand, reported that her immediate family did not routinely serve traditional foods: “I guess it depends on the family… we usually have it [corn soup] on birthdays and stuff like that… sometimes within a week but not a lot”.

Though most participants did not regularly consume traditional foods in their homes, family and loved ones were often those that they shared traditional food experiences with. Melody told us: “well me and my dad really like to go picking strawberries.” Margaret shared how she and her partner would engage in cultural activities together. She explained: “he [my boyfriend]’s trying to get back like into longhouse traditions and stuff so when he comes down that’s what we do.” Two women told stories of having traditional food experiences with their extended family members. In particular, they both shared stories of baking traditional foods with their grandmothers. Maria stated: “I’ve helped cook ‘em like especially at my grandma’s when she would be making whatever I’d help her make those, I’d help her make scones or just bread or the Indian cookies or Indian donuts too.” Grace shared that her traditional food consumption was often at her grandmother’s, when she said this: “my grandma makes them [traditional foods] so… I eat them there.” Both Grace and Maria also shared that traditional foods were often served at their extended family celebrations. For instance, Maria said: “and birthdays… someone will have [traditional foods]… I think I had ham and scone for my birthday last year.” Similarly, Grace shared, “so like my one cousin really likes corn soup so she’ll be like ‘oh I want corn soup at my birthday.’” On the other hand, two participants reported having limited connection to their extended family and, thus, did not have stories of traditional food experiences to share outside of their immediate family. Margaret explained this when she said: “we don’t really have other family right so we just… it’s always just us”.

## 4. Discussion

Overall, participating female youth demonstrated unique understandings of what traditional foods encompassed. The most commonly identified traditional foods included corn soup, game and deer meat, strawberries, and mush. These foods align with the frequently used definition of traditional foods as being those that are acquired from the local natural environment [6,7,8]. While a number of foods were collectively identified as traditional, there was also diversity in how participants classified traditional foods. Several participants even struggled with the tension of what it meant for foods to be “traditional.” In the literature, there has also been some contention with regards to how traditional foods are defined. For example, some participating Indigenous adults in a recent food Photovoice study considered traditional foods to be only those that existed pre-settler contact, while others classified foods as traditional if they had been served in their family for generations [1]. It could be the case that the traditional food understandings among First Nations youth have been shaped by the foods that have been served by their homes, thus resulting in unique perceptions of traditional foods. It is also possible that some youth viewed both pre- and post-settler foods as traditional, as these items have always been part of community events in their lifetime. On the other hand, some authors argue that more recent traditional food inventions may actually undermine Indigenous food sovereignty and advocate against classifying foods prepared using Western ingredients as traditional [14]. Western ingredients include those that were first introduced by European settlers. These foods were often imposed upon Indigenous Peoples in the form of government-issued rations, intended to “deculture” them [14]. Of note, the foods that were less often classified as traditional among participating youth were those that feature Western ingredients. However, other researchers, such as Luppens and Power [1], have argued that the creation of new cultural dishes using Western ingredients demonstrates the adaptability and resilience of traditional food systems to respond to a rapidly changing food environment. It was evident that participants had different viewpoints on what “traditional food” meant as well.

Interestingly, this diversity in how traditional foods were classified also applied to their nutritional benefits. Two youth identified that the nutritional value of traditional foods could vary based on the ingredients they were made with and how they were prepared. In the literature, the nutritional distinction between “original” versus more recent traditional food inventions has also been noted. Most existing studies investigating traditional food consumption have classified traditional foods as only those available pre-settler contact, which is, at least, in part due to the nutritional superiority of these foods acquired from the land [4,6,15,16,17]. Some authors, such as Luppens and Power [1], advocate for the recognition of the cultural value foods can hold, regardless of their nutritional quality. It is likely also the case that the participants in the present study who did view more recent inventions as traditional foods had experienced the value of these items for their culture and community.

Another key finding was that participants generally enjoyed traditional foods, but that most had limited traditional food experiences and knowledge. Preference has been identified as an important determinant of traditional food consumption in several other studies [3,4,29]. However, while preference is important, there are clearly other factors at play that influence traditional food choice. In this study, despite collective enjoyment of traditional foods, all participants but one reported that they did not consume traditional foods on a regular basis. Similarly, Genuis and colleagues [29] also identified that preference was a determinant choice among Indigenous children, but that many foods that participants enjoyed, including traditional foods, were not captured in their photos, because they were not available at home. The limited consumption of traditional foods among participants in the present study also aligns with the general recognition that traditional food consumption has declined over time, and that Indigenous Peoples living in more southern areas tend to consume less traditional foods [3,7,9]. In addition, the low consumption of traditional foods among youth participating in this study is congruent with the common finding that traditional food intake increases with age [1,9,17,20]. As has been identified in numerous studies, there are many barriers to accessing traditional foods [2,3,4,7,20]. It is likely also the case that female youth did not regularly consume traditional foods, due to a number of constraints.

In addition to limited traditional food consumption, participating youth reported even more limited experience in gathering and preparing traditional foods. In the present study, participants’ limited experiences with traditional foods could likely be associated with them having limited traditional food knowledge, which may be one contributing factor to their infrequent consumption of these foods. Limited traditional knowledge has been widely cited as a significant barrier to traditional food access and consumption [1,3,4,7]. Several young people identified that they enjoyed learning about traditional foods, but that there were limited opportunities to do so. Intergenerational relationships and contact with elders have been identified in the literature as crucial for passing on traditional knowledge to younger generations [1,3,11,13]. However, researchers have also reported that traditional knowledge has declined over time as a result of both reduced opportunities for knowledge-sharing, as well as reduced overall traditional food consumption [1,3,4,7].

Another important finding in this study was that there was a collective understanding and appreciation of traditional foods among community members living on the reserve. In fact, for several participants in this study, community events and celebrations were the main place where they consumed and experienced traditional foods. In their literature review on urban Indigenous food security, Skinner and colleagues [11] also found that Indigenous youth often had limited overall access to traditional foods, with community events being the only exposure for some. Similarly, in their ethnographic study in two First Nations communities in Northern Ontario, Robidoux et al. [13] found that community events were an important time for younger generations to learn about traditional food practices.

Another point of interest is how participants identified that, while traditional foods were not regularly consumed in most of their homes, they were still an important part of their community culture and collective knowledge. This finding relates to the idea that most Indigenous Peoples within Canada no longer rely on traditional foods as their main source of sustenance or food security, particularly in southern contexts, similar to where the present study took place, where market foods are readily available and affordable [13]. Despite not being required for survival, traditional foods remain valuable in terms of their role in cultural and community connection [9,13].

In this study, two participants also identified that attending traditional elementary schools in the community played a significant role in their learning and knowledge of traditional food practices. With a number of elementary schools located on-reserve, not all children would receive this traditional education in their schooling. In addition, since the participating community is in close proximity to a number of nearby cities, the vast majority of youth attend high school in a neighbouring city. Other studies have found that schools have the opportunity to enhance knowledge of and access to traditional foods. For example, elementary and high school traditional food programs in both rural Saskatchewan and Inuvialuit Settlement Regions have demonstrated success in providing children and youth with opportunities to experience and learn about traditional foods [30,31].

Finally, another major theme that arose was the influence of family on traditional food access and experiences. Participants identified that their immediate family and home environments were particularly important, as youth were in most cases reliant on their parents for the provision and procurement of meals and food. The formative impact that parents and caregivers have on the diets and food preferences of their children has been discussed extensively in the literature [1,3,28,29]. While parents have the opportunity to expose their children to traditional foods and learning opportunities, there may be a number of barriers to doing so, such as time constraints, access to land, lack of knowledge of skill, and cost [2,3,7].

Of the limited traditional food experiences participants did have, many were reported to be with family. In particular, two women reported experiences preparing traditional foods and learning from their grandmothers. The role of grandparents and Elders has been identified in the literature as a common source of traditional knowledge for younger generations [13,31]. Several participants also spoke of extended family gatherings and celebrations as times when traditional foods were served. This further denotes important role of traditional foods in enhancing cultural and social connection. Two women, however, reported limited connection to their extended family, which could be a factor that contributed to their limited consumption of traditional foods. Studies exploring traditional food access in urban contexts have found that disconnection from family is a significant barrier to traditional food consumption [2,7,11].

While some of the findings in this study are comparable to existing research, this study helps grow the small body of evidence on the determinants of traditional food consumption. It also offers novel insights, through involving the unique population of First Nations female youth living on-reserve in an urban context, and using Photovoice and CBPR methodologies to explore their traditional food experiences. One of the strengths of this study is that it is the first to use Photovoice and CBPR methodologies to explore the perceptions and experiences of traditional food among Indigenous youth within Canada. As a methodology, some primary strengths of using Photovoice are that it helps bring visibility to community-identified issues, provides participants with the autonomy to choose the subject and meaning of their photographs, and allows for the collection of rich and detailed information [22,24]. CBPR has been recognized as a decolonizing methodology, as it prioritizes community members as the guiding voices of the research process, in order to ensure studies are of benefit to the communities involved [22,23]. Each stage of this study has been directed by community members, who have been partners and champions of this work. Furthermore, the findings from this study will provide information, to be considered in the development of relevant community programming and resources.

A primary limitation of this study, that should not be overlooked when interpreting our findings, was its small and non-diverse sample size. Despite a variety of recruitment efforts that took place from December 2018-July 2019, only five young people were recruited to participate, all of whom self-identified as female. One possible explanation for these challenges in recruiting participants is that Photovoice requires a significant time commitment from participants, especially in comparison to most other research methods. In addition, this study is the first phase of a broader community research project taking place over the next few years. Being the first component of this project could have impacted recruitment as it takes time to build community awareness and interest. In addition, all members of the research team, who were involved in participant recruitment, self-identified as female, which may have contributed to only female youth being recruited. Due to the small sample size and lack of diversity, the results of this study cannot be generalized to all First Nations youth living in the community where this study took place. Despite these limitations, these findings still provide important insights into the food experiences and choices of this population, as well as a foundation on which this broader research project can continue to grow.

## 5. Conclusions

This is the first Canadian study to explore the perceptions and experiences of traditional foods and knowledge among First Nations youth through Photovoice. From participant interviews and photos, four themes pertaining to traditional foods were identified. In particular, participants’ understandings of traditional foods were contextual and informed by their family and community experiences. While all female youth identified a preference for traditional foods, they described having limited experiences related to acquiring, growing, and preparing traditional foods. The majority of youth also reported limited habitual traditional food consumption. Another important finding was that there was a collective understanding and appreciation of traditional foods in the community. In fact, for several participants in this study, community events and celebrations were the main place where they consumed and experienced traditional foods. Finally, the family context influenced participants’ perceptions and experiences of traditional foods. Four of the five youth reported that traditional foods were not habitually served in their homes. However, many of the traditional food experiences described by participants involved their family members or loved ones.

The findings suggest that it would be beneficial to explore opportunities to offer traditional food literacy education for First Nations youth. Two participants described how the majority of their traditional food experiences and knowledge came from attending a traditional elementary school on-reserve. As such, it would be worthwhile to explore how all schools on-reserve could better integrate traditional food education into their curriculums. The social environments of youth are also important to consider while exploring traditional food skills education opportunities. In particular, it would be worth exploring opportunities to offer food skills activities in existing youth programs in the community, as these provide an ideal social setting for peer learning. Another option would be to expand the existing food programming offered in the community to engage more youth as participants. As families had a significant influence on participants’ traditional food consumption and experiences, the engagement of family must also be taken into account. Family-based education has been suggested by others, such as Neufeld and colleagues [3]. Furthermore, the broader community was described as having an important influence on participants’ perceptions and experiences of traditional foods, with the majority of participating youth primarily accessing traditional foods at community events. Given the importance of community described, it would be of benefit to explore opportunities to expand existing programming that connects community members through food.

Continued community-based research, exploring the perceptions and experiences of traditional foods and the determinants of traditional food consumption among Indigenous groups living in southern and urban contexts is recommended. This study can serve as an example of how to effectively explore the traditional food experiences of Indigenous youth. The methodologies and methods employed in this study could be adapted and used to explore the determinants of traditional food choice among Indigenous individuals, and youth in particular, in similar or varying geographical contexts.

The results of this study will be disseminated in the future, in conjunction with additional findings from the broader research project. Next steps for the research team involve recruiting five additional youth to participate in this project. If possible, involving a research team or community member who self-identifies as male in further participant recruitment efforts will be considered, in order to better support the recruitment of male youth. Following their participation, an elder-led sharing circle will be organized for the 10 youth participants to attend. Consideration will be given to additional strategies that would better support the recruitment of a diverse sample of participants. Events and activities to disseminate the study findings to the community at-large will be determined, in consultation with the Community Advisory Group. It is expected that these efforts will involve activities such as a community feast and a photo exhibit. Community partners will be invited to co-present study findings at conferences and events, whenever possible. The overall findings of the broader research project will be used to inform the development of local resources and programs aimed at promoting traditional food consumption, through enhanced social supports and sharing traditional knowledge.

In conclusion, this study adds to the literature in a number of ways. In particular, this study is the first to use Photovoice methodology to explore the perceptions and experiences of traditional foods among Indigenous youth in urban and southern regions. It is one of the few existing studies to explore the perceptions and determinants of traditional food consumption in the unique geographical context of an on-reserve community in southwestern Ontario. Exploring social avenues to enhance traditional food skills and knowledge among youth in the community is recommended. In addition, future community-engaged research that explores the determinants of traditional food choice is suggested, in order to add to a small but growing body of evidence in this important research area.

## Figures and Tables

**Table 1:** Study Participants.

Participant Pseudonym	Gender	Age	Educational Background
Maria	Female	15	High school student
Grace	Female	15	High school student
Melody	Female	15	High school student
Georgia	Female	16	High school student
Margaret	Female	22	College student
